# Research Progress and Challenges in Vaccine Development against Classical Swine Fever Virus

**DOI:** 10.3390/v13030445

**Published:** 2021-03-10

**Authors:** Qiang Wei, Yunchao Liu, Gaiping Zhang

**Affiliations:** 1 Henan Provincial Key Laboratory of Animal Immunology, Henan Academy of Agricultural Sciences, Zhengzhou 450002, China; chrysqiang@126.com (Q.W.); yunchaoliu2012@163.com (Y.L.); 2 College of Animal Science and Veterinary Medicine, Henan Agricultural University, Zhengzhou 450002, China

**Keywords:** classical swine fever, DIVA concept, vaccine, efficacy, safety

## Abstract

Classical swine fever (CSF), caused by CSF virus (CSFV), is one of the most devastating viral epizootic diseases of swine in many countries. To control the disease, highly efficacious and safe live attenuated vaccines have been used for decades. However, the main drawback of these conventional vaccines is the lack of differentiability of infected from vaccinated animals (DIVA concept). Advances in biotechnology and our detailed knowledge of multiple basic science disciplines have facilitated the development of effective and safer DIVA vaccines to control CSF. To date, two types of DIVA vaccines have been developed commercially, including the subunit vaccines based on CSFV envelope glycoprotein E2 and chimeric pestivirus vaccines based on infectious cDNA clones of CSFV or bovine viral diarrhea virus (BVDV). Although inoculation of these vaccines successfully induces solid immunity against CSFV, none of them could ideally meet all demands regarding to safety, efficacy, DIVA potential, and marketability. Due to the limitations of the available choices, researchers are still striving towards the development of more advanced DIVA vaccines against CSF. This review summarizes the present status of candidate CSFV vaccines that have been developed. The strategies and approaches revealed here may also be helpful for the development of new-generation vaccines against other diseases.

## 1. Introduction

Classical swine fever (CSF) is a highly contagious, economically significant, multi-systemic viral disease in swine [1], which is notifiable to the World Organization for Animal Health (OIE). The causative agent, CSF virus (CSFV), is an enveloped, positive-sense, single-stranded RNA virus. It belongs to the *Pestivirus* genus within the Flaviviridae family [2]. Other members of this genus are bovine viral diarrhea virus (BVDV) 1 and 2, border disease virus (BDV), and an increasing number of tentative pestivirus species [3,4]. According to the recent reclassification of *Pestivirus* genus by the International Committee on Taxonomy of Viruses (ICTV), BVDV-1 is re-designated as Pestivirus A, BVDV-2 is re-designated as Pestivirus B, CSFV is re-designated as Pestivirus C, BDV is re-designated as Pestivirus D, and many of tentative *Pestivirus* species are re-designated as Pestivirus E-K [5].The CSFV genome encodes a precursor polyprotein of 3898 amino acids (aa), which is post-translationally processed into four structural proteins (C, E^rns^, E1 and E2) and eight non-structural proteins (N^pro^, p7, non-structural protein [NS] 2, NS3, NS4A, NS4B, NS5A, and NS5B) by cellular and viral proteases [6,7]. Antibodies against the envelope glycoproteins E2, E^rns^, and NS3 have been detected in pigs that recover from infection [8,9]. The major immunogen is the glycoprotein E2, in terms of inducing neutralizing antibodies and protection against future infection [9,10].

CSF has consistently caused important economic losses to the pig industry since its emergence [11,12]. Several countries have succeeded in eliminating CSF following the implementation of strict control measures. However, in most parts of the world with significant pig production, CSF is at least sporadically present. To date, CSF remains endemic in South and Central America, Eastern Europe, and neighboring countries, and Asia. Little is known about the African situation. The most recent outbreaks have been reported in Korea, Colombia, Russia, Brazil, and Japan [13]. In particular, the reemergence of CSF from previously CSF-free Japan in September 2018 has caught much attention [14,15]. Thus, CSFV remains an endemic and reemerging virus in pigs and continues to threaten pork production worldwide and the food security of populations in developing countries.

Systematic vaccination and non-vaccination stamping-out are the two main strategies to control CSF [16,17]. In CSF-free zones, or where eradication is in progress, control is based on the non-vaccination and strict stamping-out policy. In contrast, due to the enormous costs of stamping-out, systematic prophylactic vaccination is a more effective strategy for CSF control in CSF endemic countries. As with many other viral infections affecting livestock, several highly efficacious live attenuated CSF vaccines have been widely used for decades and have paved the way to successful eradications in many areas. However, the use of these vaccines interferes with the serological diagnosis, as they lack differentiability of infected from vaccinated animals (DIVA). Indeed, due to the trade restrictions that are imposed on pigs vaccinated with conventional live attenuated vaccines, only DIVA vaccines are considered a feasible option for future control and eradication of CSF. Therefore, the development of potent DIVA vaccines poses a challenge for research groups worldwide. So far, different concepts have been investigated during the development of CSFV DIVA vaccines, including vector vaccines, recombinant attenuated vaccines with chimeric constructs, subunit vaccines, peptide vaccines, and RNA/DNA vaccines. This review is aimed at describing progress and challenges associated with CSFV vaccine development, revealing the strategies and approaches that may also be helpful for the development of next-generation vaccines against other pathogens.

## 2. Conventional Live Attenuated Vaccines and Their Application

In the early 20th century, the primary vaccines against CSF were developed, which consist of the virus and porcine hyperimmune serum, followed by the crystal-violet vaccine [18]. However, the safety and efficacy of these vaccines were poor. Further investigations aimed at the development of live attenuated vaccines were performed in rabbits since the 1940s [19,20]. During that time, the Chinese vaccine strain (C-strain), the so-called Chinese hog cholera lapinized virus (HCLV), was widely used in both mainland China and many other countries due to its better safety and efficacy than other strains [21]. In addition to passage through rabbits, researchers also tried other methods to attenuate CSFV. For instance, the low-temperature-adapted Japanese guinea-pig exaltation-negative (GPE^−^) strain [22,23] and the French cell culture-adapted Thiverval strain were obtained [24,25]. Nowadays, the most commonly used strains are the C-strain, the lapinized Philippines Coronel (LPC) strain, the GPE^−^ strain, the Thiverval strain, and their derivative strains [26,27].

The C-strain was developed jointly by the China Institute of Veterinary Drugs Control and the Harbin Veterinary Research Institute in China in 1956 [28]. It was developed by attenuating a highly virulent strain through at least 480 passages in rabbits [28]. The strain spread worldwide. At present, many commercial strains were derived from the C-strain, such as Pestiffa (French), VADIMUN (USA), and Riems (Germany), which have also been widely used. The C-strain formulations used in both China and Europe indicate that reliable protection is provided as early as 72 or 96 h after a single vaccination [29,30,31]. Immunity can persist for at least 6–18 months and even be lifelong [30,32]. Studies targeting the genetic stability of the C-strain proved that the vaccine is highly stable [30]. In addition, the C-strain is safe in young piglets or pregnant sows, even in immunosuppressed individuals [33]. In addition, inoculation of the vaccine confers complete protection against CSFV isolates from all three genotypes [34]. Likewise, the commercial LPC, GPE^−^, and Thiverval vaccines show similar performance as C-strain vaccines.

In addition to the wide use of C-strain as injection vaccine for most domestic pigs, it can also be used as oral vaccine for wild boars and domestic pigs in rural areas [35,36]. Indeed, oral vaccination with the C-strain was proved to be safe and effective [37,38]. Recently, an oral formulation of the C-strain was improved. Researchers have reported oral baits obtained by absorption of C-strain onto bread followed by lyophilization. While the current commercial oral vaccine bait containing liquid C-strain vaccine requires storage at −20 °C, this new oral bait is stable for 18 months at 4 °C. Pigs vaccinated with these new oral baits displayed seroconversion after 14 days [39]. Thus, the new formulation provides a more cost-effective method to improve vaccination of domestic pigs and wild boars.

The requirements of the ideal vaccine postulated by Terpstra and Kroese [40] mainly regard safety, efficacy, and marketability. For CSFV, live attenuated vaccines have long been considered to meet all the requirements. Nevertheless, a recent outbreak that occurred in a previously CSF-free island applying live attenuated low-virulence strain of Miyagi (LOM) vaccine inoculation led to a later study showing that the employed LOM strain can cause viremia and cross the placenta to piglets [41,42]. This research reminds us of the need to recharacterize the safety of the live attenuated vaccines using more recent technologies. In addition, another main drawback of the live attenuated vaccines is the lack of a serological marker concept [26,43,44] that would allow differentiation of infected from vaccinated animals (DIVA concept). Due to the trade restrictions that are imposed on pigs vaccinated with conventional live attenuated vaccines, the development of novel potent vaccines combined with the DIVA concept becomes more and more important.

## 3. Live Attenuated DIVA Vaccines

Vaccines that allow easy but accurate serological differentiation between infected and vaccinated animals are called marker vaccines. These vaccines are developed to meet all demands regarding safety, efficacy, DIVA potential, and marketability. Therefore, marker vaccines can also be called DIVA vaccines [45]. Since the early 1990s, researchers have made many attempts to develop new DIVA vaccines against CSFV. One of the most promising trials is the development of live attenuated DIVA vaccines (Table 1).

### 3.1. Chimeric Pestivirus Vaccines

The first strategy in the development of candidate CSF DIVA vaccines is the development of a recombinant chimeric vaccine. Among the most promising candidates are chimeric pestiviruses based on the infectious cDNA clones of CSFV or BVDV [46,47,48,49].

So far, several chimeric pestiviruses have been developed, and some of them have been extensively studied in the target species, such as “flc11” [50], “flc9” [51,52], and “CP7_E2gif” [53,54]. However, only two vaccines have been licensed until now. In Europe, a chimeric pestivirus named “CP7_E2alf” (Suvaxyn® CSF Marker, Zoetis) was licensed as a DIVA vaccine in 2014. In South Korea, a chimeric pestivirus designated Flc-LOM-BE^rns^ was licensed in 2017.

The chimeric pestivirus “CP7_E2alf” DIVA vaccine was constructed using the infectious cDNA clone of the cytopathogenic BVDV strain “CP7”, in which the E2 gene was replaced with that of CSFV strain “Alfort/187” [46,55,56]. The evaluation of the “CP7_E2alf” vaccine for both intramuscular and oral application was performed in detail. The data with regard to the construction, genetic stability, safety, efficacy, DIVA diagnostics, and strategy design are generally available [52,57,58,59,60,61,62]. In brief, multiple studies have demonstrated that “CP7_E2alf” is as safe and efficacious as the conventional live attenuated vaccines [62]. However, the researchers mentioned that transplacental transmission in pregnant animals could not be prevented in some cases when an early and highly virulent challenge was carried out to evaluate the suitability for emergency vaccination scenarios [32]. Therefore, the summary of product characteristics included a warning, which states that sows should not be vaccinated due to the risk of birth of persistently infected offspring [63]. A more recent research was performed to add to the safety and efficacy studies of the vaccine. In this study, a single-dose vaccination of “CP7_E2alf” in sows, which were challenged with a moderately virulent CSFV strain 21 days post-vaccination (dpv), could efficiently protect the offspring. This result indicated that transplacental transmission of a moderately virulent CSFV was completely prevented [61]. In addition, the main drawback of the “CP7_E2alf” vaccine is the cross-reactivity observed with sera from BDV- and BVDV-infected animals in the E^rns^-based differential ELISAs [62,64]. Reduced specificity was also observed in piglets with maternally derived antibodies (MDAs) induced by vaccination of sows with the “CP7_E2alf” vaccine, which would complicate the serological surveillance [65].

The other licensed chimeric pestivirus vaccine, designated Flc-LOM-BE^rns^, was developed based on the live attenuated CSFV vaccine strain LOM [66]. In this chimera, the 3′ end capsid gene and the complete E^rns^ gene were replaced by the corresponding sequences of KD26 BVDV strain. Like the “CP7_E2alf” vaccine, the Flc-LOM-BE^rns^ vaccine showed high efficacy. Furthermore, the vaccine could protect fetuses from transplacental transmission after challenge of pregnant sows with CSFV strain YC11WB in three different pregnancy periods (early, mid, or late). However, since vaccination with this chimeric vaccine started in Korea in 2020, comprehensive data about the safety of the vaccine and the robustness of the DIVA concept are still lacking. Nevertheless, the DIVA capacity of the two chimeric pestivirus vaccines (“CP7_E2alf” and Flc-LOM-BE^rns^) is the same, which is based on the detection of antibodies against CSFV E^rns^ being indicative of a field virus infection. Thus, cross-reactivity is also likely to occur for Flc-LOM-BE^rns^, although this is not reported in the limited number of pigs [66].

Chimerization of closely related viruses could facilitate viral replication, but at the same time may be problematic in terms of induction of the production of cross-reactive antibodies. A recent study posed the hypothesis that genetically distinct pestiviruses might create replicating chimeric viruses with improved DIVA capacity due to a significantly reduced risk of inducing the cross-reactive antibodies [67]. In this study, the chimeric pestivirus vaccine candidates were generated using the cDNA clone of CSFV strain Alfort-p447, in which the E^rns^ encoding sequence was replaced by the homologous sequences from pestiviruses distantly related to CSFV. Consequently, a “Ra” chimera containing the E^rns^ gene of Norway rat pestivirus (NrPV) isolate NYC-D23, a “Pro” chimera harboring the E^rns^ sequence of Pronghorn antelope pestivirus, and a “RaPro” chimera combining parts of both E^rns^ sequences were generated. The results demonstrated that the CSFV genome can tolerate the replacement of the E^rns^ gene by the distantly related sequences. The three chimera conferred solid protection at 28 dpv, representing a later onset of immunity compared with “CP7_E2alf” [62]. In contrast, cross-reactivity was not detected in the sera of all vaccinated pigs using the CSFV E^rns^ antibody-based DIVA assay, representing an improved DIVA capacity. Therefore, development of chimeric pestivirus vaccines containing E^rns^ sequences of distantly related pestiviruses is a very promising strategy for establishing an improved serological negative marker in CSF DIVA vaccines. Further studies, including the evaluation of the safety, efficacy, and robustness of the DIVA concept, are needed to provide more valuable data of these candidates.

### 3.2. Recombinant Deletion or Mutation DIVA Vaccines

By partial or complete deletion of a gene, the mutant strain is probably highly attenuated and simultaneously provides a “negative marker” [27]. A promising trial was the development of a CSFV DIVA vaccine candidate, with both positive and negative antigenic markers called FlagT4Gv [68]. In this candidate, the highly conserved monoclonal antibody (mAb) WH303-recognized epitope (^829^TAVSPTTLR^837^) of the E2 glycoprotein was eliminated and a Flag epitope was included in E1 as a positive marker. The inoculation of FlagT4Gv could induce immunity against challenges as early as 3 dpv [69]. Other rational designs were also proposed and needed further investigations, such as the alterations in fusion peptide regions of E2 [70], codon de-optimization in the E2 encoding region [71], and the exchange of hypervariable regions in the E2 protein [72].

Indeed, as stated by Kortekaas [73], a maximum safe marker vaccine would ideally arise from a potent and safe vaccine strain by mutations in only one antigenic domain or even a single epitope. As mentioned above, most attempts to constructing this kind of CSFV marker vaccine mainly focus on the mutation of the mAb WH303-recognized epitope of the E2 glycoprotein. However, this epitope is a highly conserved and immunogenic epitope of CSFV, which induces the production of neutralizing antibodies [17,74]. Thus, mutation of this epitope would probably affect immunogenicity.

In a very recent study, the CSFV HQ06-recognized epitope (^116^LFDGTNP^122^) was chosen to construct a DIVA vaccine [75]. This epitope is a conserved linear epitope, but its ability to induce the production of neutralizing antibodies is weak [76], thus its mutation does not affect the immunogenicity of the virus. At first, three mutant viruses harboring the single aa mutation at ^117^F, ^119^G, or ^122^P, designated as rHCLV-E2F117A, rHCLV-E2G119A, and rHCLV-E2P122A, were constructed based on the backbone of the C-strain. Subsequently, animal experimental results of rHCLV-E2P122A showed that the variant retained the characteristics of the C-strain in both rabbits and pigs, but the antibodies induced by this vaccine lost reactivity with the mAb HQ06-recognized epitope. Therefore, rHCLV-E2P122A may have the potential to be developed as a CSF DIVA vaccine candidate. However, the method of detecting the antibodies against the HQ06-recognized epitope is not commercial, revealing that DIVA tests based on the negative marker are needed.

## 4. Viral Vector Vaccines

Since the early 1990s, the application of viral vectors for immunization against CSF has been reported [77,78]. Until now, viral vectors used in candidate CSF DIVA vaccines include swinepox virus (SPV), pseudorabies virus (PRV), and adenovirus (AdV). Induction of a protective immune response relies on virus-neutralizing antibodies against E2 protein, revealing its role as a major immunogen. Thus, in most cases, the viral vectors expressed CSFV E2 (Table 1). In addition, the detection of NS3-specific antibodies is less suitable for CSFV-specific serological marker testing because of the high genetic stability of the NS3 protein and the prevalence of non-CSFV pestiviruses in swine. Therefore, the most promising DIVA strategy is based on detection of E^rns^ -specific antibodies in infected swine [44,64].

### 4.1. Swinepox Virus Vector

SPV possesses the ability to carry and express large amounts of foreign genetic material due to its large double-stranded DNA genome. The virus infects only pigs. The infection is typically mild and occasionally accompanied with localized skin lesions that heal naturally [79]. Lin et al. constructed a recombinant rSPV-E2 vaccine candidate using the SPV genome as the backbone, in which the gene encoding glycoprotein E2 was inserted by homologous recombination [80]. Intramuscular immunization of rSPV-E2 induced a substantial CSFV-specific neutralizing antibody response in pigs and provided clinical protection against CSFV challenge. Therefore, the recombinant rSPV-E2 may be a promising candidate vaccine against CSFV infection. To further obtain a safer and more efficient SPV vector, the researchers constructed three SPV mutants, Δ003, Δ010, and ΔTK and performed biosafety assessments both in vitro and in vivo [81]. Except for inoculation with high titers of Δ010, which caused slight skin inflammation, all other experimental results have demonstrated that the SPV mutants Δ003 and ΔTK may be promising candidates for an attenuated viral vector in veterinary medicine. Improvements in the efficacy and safety of these mutants in the practical application of viral vector vaccines against CSFV are expected.

### 4.2. Pseudorabies Virus Vector

Several proteins have been confirmed to be associated with the virulence of PRV, such as gE, gI, thymidine kinase (TK), and protein kinase (PK) [82,83]. The corresponding genes could be replaced with foreign genes without affecting the infection and propagation of the virus. Thus, PRV is often used as a vector to express major immunogens of other viral pathogens to develop multivalent vaccines. As early as 1991, PRV vectors that express the gene encoding E2 of CSFV have been constructed and found to induce protective immunity [78]. Since then, investigations into the use of PRV vectors as candidate DIVA vaccines against CSFV have continued. Previously, two recombinant viruses rPRVTJ-delgE [84] and rPRVTJ-delgE/gI/TK [85,86], were generated by a research team. rPRVTJ-delgE is a gE/gI-deleted PRV mutant based on the emergent PRV variant. The gene encoding TK was further deleted to generate the gE/gI/TK-deleted mutant rPRVTJ-delgE/gI/TK. Subsequently, the researchers generated the recombinant PRV variants rPRVTJ-delgE/gI-E2 and rPRVTJ-delgE/gI/TK-E2 expressing the E2 glycoprotein based on the mutant rPRVTJ-delgE and rPRVTJ-delgE/gI/TK, respectively [84,85,86]. The immunogenicity and efficacy of the variants in pigs were evaluated. The results showed that rPRVTJ-delgE/gI-E2 and rPRVTJ-delgE/gI/TK-E2 were both safe for pigs. They could induce detectable neutralizing antibodies against CSFV and PRV. Furthermore, they provided solid protection against the lethal challenge with either the CSFV Shimen strain or the PRV TJ strain. These findings suggest that the two recombinant PRV variants are promising bivalent DIVA vaccine candidates against CSFV and PRV coinfections. A trivalent variant rPRVTJ-delgE/gI/TK-E2-Cap was constructed later, which expresses the E2 protein of CSFV and the Cap protein of PCV2 [87]. The results showed that inoculation in rabbits and pigs induced detectable PRV-specific neutralizing antibodies. However, antibodies against E2 and Cap were not detected. Therefore, further research is desirable to optimize the design of rPRVTJ-delgE/gI/TK-E2-Cap, which could serve as a trivalent DIVA vaccine candidate.

Very recently, a new bivalent PRV/CSFV DIVA vaccine candidate JS-2012-ΔgE/gI-E2 was described [88]. The recombinant vaccine candidate was developed by inserting the E2 gene into the gE/gI-deletion PRV variant strain JS-2012-ΔgE/gI. The results showed that the recombinant virus was safe for piglets. A single vaccination of JS-2012-ΔgE/gI-E2 was sufficient to provide complete protection to piglets against lethal challenge of PRV variant and CSFV.

CSFV and PRV infections remain the most significant threats to the pig industry in many countries, especially in China. The bivalent vaccines against both CSFV and PRV could simplify the vaccination programs and minimize the cost. In addition, a commercial gE-ELISA kit is available. Meanwhile, an ELISA method has been developed for the detection of CSFV E^rns^ antibodies [64]. Thus, combined with gE- and E^rns^-ELISA, the recombinant PRV variants containing the gene encoding E2 are promising bivalent DIVA vaccine candidates that might play an important role in the control and eradication of both CSFV and PRV.

Nevertheless, PRV has been eliminated from domestic pigs in some countries, and PRV vaccines were prohibited. Thus, the use of these candidates should be limited. In addition, the C-strain and Bartha-K61 are widely used in China, and almost all young piglets carry CSFV and PRV MDAs. As revealed in the evaluation of JS-2012-ΔgE/gI-E2 [88], the PRV MDAs could severely interfere with the humoral responses to vaccination, which is a major barrier to the practical application of PRV vector vaccines. Therefore, the priming-boosting strategies should be performed to overcome interference by MDAs.

### 4.3. Porcine Reproductive and Respiratory Syndrome Virus Vector

Recently, a series of studies on a recombinant vaccine rPRRSV-E2 were carried out by Gao et al. [89,90,91]. The rPRRSV-E2 was constructed by reverse genetics. It was generated by inserting the CSFV gene encoding E2 into the backbone of HuN4-F112, which is an attenuated vaccine strain for highly pathogenic PRRSV (HP-PRRSV). A preliminary study showed that single-dose immunization with rPRRSV-E2 could induce the production of PRRSV-specific and CSFV-specific antibodies and fully protect pigs from lethal challenge of HP-PRRSV and CSFV [89]. A subsequent study revealed that MDAs against either PRRSV or CSFV did not interfere with the immunity of rPRRSV-E2 [90]. Very recently, it was demonstrated that rPRRSV-E2 confers long-term immunity against HP-PRRSV and CSFV, which maintains for at least 20 weeks after vaccination [91].

PRRSV MDAs did not interfere with the immunity of rPRRSV-E2, which might result from relatively lower neutralization titers of the PRRSV MDAs for rPRRSV-E2 [90]. In addition, the long period of immunity conferred by rPRRSV-E2, which almost corresponds to the expected lifespan (18–26 weeks) of commercial pigs, will have significant social and economic benefits. Taken together, rPRRSV-E2 is a promising bivalent DIVA vaccine candidate against both PRRSV and CSFV infection and requires only a single vaccination in swine.

### 4.4. Adenovirus Vector

Recombinant adenoviruses are versatile tools for gene delivery and expression. At first, only porcine adenoviruses (PAdVs) were used as vectors to express the CSFV E2 protein, but the immunization results were not always as good as expected [27]. With more data available about the efficacy of human AdV5 vaccines against different pathogens [92,93,94,95,96], the recombinant replication-deficient AdV5 represents a promising vector for the development of human and animal vaccines. Sun et al. developed an adenovirus-delivered, alphavirus replicon-vectored vaccine against CSF (rAdV-SFV-E2) [97]. Comprehensive studies with this candidate were carried out and the results were reviewed [32]. Overall, the results showed that [97,98,99,100,101]: (1) the vaccine was safe for mice, rabbits and pigs; (2) two-dose immunizations with the vaccine as low as 6.25 × 10^5^ TCID_50_ or a single-dose immunization with 10^7^ TCID_50_ rAdV-SFV-E2 provided complete protection against a lethal CSFV challenge in pigs; (3) MDAs to either rAdV-SFV-E2 or the C-strain did not interfere with the efficacy of the vaccine; (4) inoculation of rAdV-SFV-E2 did not trigger interference with anti-vector immunity; (5) co-administration with a live PRV vaccine has no effect on vaccine efficacy; (6) MDAs from the sows immunized with the vaccine are sufficient to confer complete passive immunity to newborn piglets; and (7) a *Salmonella enteritidis*-derived bacterial ghosts (BG) is a promising adjuvant to enhance the efficacy of rAdV-SFV-E2. In summary, the rAdV-SFV-E2 represents a promising DIVA vaccine candidate for control and eradication of CSF [28].

### 4.5. Other Viral Vectors

Newcastle disease virus (NDV) is an especially useful vector for expression of foreign proteins because of its unique properties. For example, the use of embryonated chicken eggs to generate large amounts of recombinant NDV (rNDV) could be an efficient and economical way to produce a vaccine for pigs. In addition, the NDV vector is also able to infect via the intranasal route, thereby inducing responses at the primary site of CSFV entry and replication. In a recent study, the rNDV strain expressing CSFV E2 and E^rns^ was constructed using reverse genetic techniques [102]. Pigs intranasally vaccinated with the vaccine showed effective humoral responses with high titers of CSFV-neutralizing antibodies, suggesting the usefulness of NDV as a vector for the development of vaccines against CSFV and other pig pathogens.

Baculoviruses would appear to be ideal gene delivery vectors for in vivo applications because they cannot replicate in mammalian cells but can efficiently deliver genes into many cell types such as human, rabbit, rodent, porcine, and bovine cells [103,104]. However, there are two bottlenecks in baculovirus-based gene delivery: complement-dependent inactivation and low transduction efficacy targeting immune cells. Thus, the efficacy of baculovirus-vectored vaccines was affected [105]. To break the bottlenecks, a recombinant virus BV-VSVG-ED-pFc-CMV-S/P-E2 was developed [106]. This novel VSV-G-pseudotyped baculovirus was constructed based on IgG1 Fc surface display. Intramuscular immunization of this recombinant virus induced high titers of CSFV-specific antibodies and neutralizing antibodies. Moreover, the level of IFN-γ secretion was improved in pigs. Thus, the design and construction of this recombinant virus offers an alternative strategy for vaccine development for CSFV and other pathogens.

## 5. Subunit Vaccines

Subunit vaccines are usually composed of only one purified immunogenic protein of the pathogen. Thus, it is easy to develop DIVA vaccines and corresponding serological diagnostic test kits with subunit vaccines. Various candidate subunit vaccines based on NS3, E^rns^, or E2 were designed and evaluated. However, so far candidate NS3 and E^rns^ vaccines were not successful [10,107]. In contrast, E2 was proved to be the most protective immunodominant protein against CSF [17]. Therefore, the E2 subunit vaccine remains the focus of CSF subunit vaccine research (Table 1). In recent years, several attempts were made to further develop E2 subunit vaccines using various approaches, such as combination with molecular adjuvant, E2 emulsion with improved adjuvant, the use of E2 protein of the epidemic strain, cost-effective E2 protein production using plant expression systems, and surface display of E2 on Gram-positive enhancer matrix (GEM) or gold nanoparticles.

### 5.1. Commercial E2 Subunit Vaccines

To date, two E2 subunit DIVA vaccines have been licensed in Europe: the BAYOVAC^®^ (Bayer AG) and the Porcilis^®^ Pesti (MSD Animal Health) [108,109]. The CSFV E2 of BAYOVAC^®^ is derived from the Brescia strain (genotype 1.2) [110], while that of Porcilis^®^ Pesti is derived from the Alfort Tübingen strain (genotype 1.1) [9]. In 2018, Tian Wen Jing (TWJ-E2^®^), the first Chinese E2 subunit DIVA vaccine against CSFV, was officially licensed and commercialized. The modified E2 glycoprotein of C-strain (genotype 1.1) is used as the immunogen of this vaccine. In summary, these vaccines are all developed based on the immunogenic E2 protein expressed in baculovirus expression systems. Serological DIVA assays, such as the *pigtype* CSFV E^rns^ antibody ELISA, are available to accompany the application of these CSFV DIVA vaccines [64].

The efficacy of BAYOVAC^®^ CSF E2 and Porcilis^®^ Pesti has been extensively assessed [9,26,110,111,112,113,114,115,116,117,118,119]. These vaccines are safe and were shown to provide clinical protection and limit the spread of CSF. However, compared with the live attenuated vaccines, the disadvantages of the two subunit vaccines are lower efficacy with a later onset of immunity, incomplete protection against transplacental transmission, and the requirement for a two-dose inoculation regime [26,120]. Therefore, these vaccines are unsuitable for emergency vaccination in CSFV-free countries (one exception being Romania), and they are also not compatible with oral delivery to wild boars. At present, only Porcilis^®^ Pesti is still commercially available.

As for TWJ-E2^®^, two-dose vaccination with this vaccine was shown to provide complete protection against the highly virulent Shimen strain. The vaccinated piglets did not display clinical signs, viremia, or pathological lesions post-challenge [121]. A recent study revealed that a prime-boost vaccination with this vaccine induced high levels of E2-specific antibodies and neutralizing antibodies, and completely protected pigs from lethal challenge by genotype 2 field strains currently circulating in China [122]. However, the onset of robust immunity against CSFV challenge elicited by two-dose TWJ-E2^®^ vaccination was much later than that induced by the C-strain due to insufficient induction of cellular immunity. This result was also observed in pigs immunized with BAYOVAC^®^ and Porcilis^®^ Pesti. In addition, both BAYOVAC^®^ and Porcilis^®^ Pesti have been reported to provide incomplete protection against transplacental transmission. Thus, the ability of the recently developed TWJ-E2^®^ vaccine to clear viruses in vaccinated pregnant sows after CSFV challenge remains to be determined.

### 5.2. Combination with Molecular Adjuvant

To generate an early response in immunized pigs, the CSFV E2 antigen was fused to the extracellular domain of porcine CD154. This E2-CD154 construct was expressed in a stable recombinant HEK 293 cell line using a lentivirus-based gene delivery system [123]. The CD154 molecule is a glycoprotein that belongs to the tumor necrosis factor superfamily. Several studies have shown that CD154 can act as a molecular adjuvant and enhance the immune responses [124,125,126]. The immunization of the E2-CD154 subunit vaccine was able to fully protect pigs from challenge as early as seven days after a single-dose vaccination. Both doses (50 μg or 75 μg) were equally effective. No clinical signs were observed in the vaccination groups. In addition, isolation of CSFV and RT-PCR were unsuccessful in blood samples and organs from pigs that had received vaccination. In order to further evaluate the efficacy of the E2-CD154 vaccine, immunization of pregnant sows with two doses of the vaccine was performed [127]. The results showed that the two-dose vaccination protected the fetuses from transplacental transmission after challenge of pregnant sows with a high dose of a highly virulent CSFV. Moreover, the humoral and cellular immune responses were both enhanced by immunization with E2-CD154 compared to immunization with E2 without CD154 in the same adjuvant formulation in mice, indicating that CD154 may act as a molecular adjuvant enhancing the immune response [128]. To further investigate the production process of E2-CD154 protein, the researchers investigated the growth and the expression profiles of the clone cells in four commercially available culture media [129]. The results suggest that the culture media CDM4HEK293 and SFM4HEK293 are the best choice to grow the HEK-293 E2-CD154 cells. In addition, the E2-CD154 protein was produced with similar conformational and immunogenic characteristics independently of the culture media, demonstrating high reproducibility and consistency among protein batches produced by HEK-293 cells. Therefore, a good basis exists for commercial production of the E2-CD154 protein. Taken together, these results suggest that the E2-CD154 vaccine might be a promising candidate for the control and elimination of CSF disease in endemic situations. Still, further studies are needed to fulfill the safety and efficacy requirements established by the OIE, including the capacity of E2-CD154 to be used for emergency vaccination [130].

In addition, since many studies have used IFNs as adjuvants to enhance protective immunity, Zhang et al. investigated whether IFN-γ as an adjuvant could improve the efficacy of E2 subunit vaccine [131]. They generated a novel construction that co-expressed the CSFV E2 and porcine IFN-γ proteins through the baculovirus system. The results showed that the new subunit vaccine containing IFN-γ significantly enhanced the specific protective immune response in weaning piglets. It will be interesting to see if this approach could prevent CSFV transplacental transmission in pregnant sows.

### 5.3. E2 Emulsion with Improved Adjuvant

Madera et al. reported another E2 subunit vaccine, KNB-E2, which contains the baculovirus-expressed E2 protein of the C-strain and an oil-in-water emulsion adjuvant, KNB [132,133]. Pigs immunized with one dose of KNB-E2 containing 75 μg of purified E2 protein can be clinically protected from CSFV challenge. To further elucidate the efficacy of KNB-E2, a series of animal studies were undertaken [134]. The results showed that a single-dose vaccination of KNB-E2 containing 25 μg of E2 protected pigs from clinical symptoms of CSF. In addition, a reduction in KNB-E2-mediated CSF symptoms was observed at 2 weeks after inoculation and clinical signs of CSF remained reduced when vaccinated pigs were challenged at two and four months after inoculation. These results indicate that the vaccine is effective in reducing clinical symptoms of CSF, suggesting that KNB-E2 has the potential to safely minimize CSF-related losses. Subsequently, a modified version of the OW-14 emulsion adjuvant formulated with the food-grade cost-effective saponin extract, Sapnov LS™ (OWq) was tested in the CSFV E2 subunit vaccine for the first time [135]. The results showed that OWq can be stored for at least 180 days with high physical and chemical stability. In addition, it was safe for use in swine immunization. Furthermore, two doses of CSF E2 subunit vaccine with OWq adjuvant induced higher total IgG and CSFV-neutralizing antibody titers in pigs compared with other emulsion vaccine prepared with oil-based adjuvant without saponins. Further investigation of this adjuvant is needed to evaluate its suitability for further development.

### 5.4. E2 Protein of the Epidemic Strain

Although insect cell systems have been used for expression of antigens for a long time, CSFV E2 expressed from this expression system is still under development for several vaccination campaigns worldwide. A VN91-E2 subunit vaccine was developed in Vietnam, which is composed of the baculovirus-expressed E2 protein of a local genotype 1.1 CSFV isolate VN91 and a Freund’s Adjuvant Complete or Incomplete Adjuvant [136]. The results demonstrated that the two-dose vaccination were effective in stimulating protective immunity against CSFV. Moreover, it can protect pigs from CSFV challenge with different genotypes (1.1, 2.1, and 2.2) that circulate in Vietnam.

In a very recent study, a novel CSFV E2 sequence (E2ZJ) was identified from an epidemic strain of Zhejiang, China [137]. To improve the production of secreted E2ZJ protein from baculovirus-infected insect cells, a signal sequence SPZJ (SP23) was selected. Expression of E2ZJ combined with the SPZJ resulted in at least a 50% increase in E2 production compared to any of the other signal peptides tested. The results of the immunofluorescence assay (IFA) and neutralization assay showed an improved immune response elicited by the E2ZJ protein compared to that induced by other E2 types. Furthermore, a single dose of 5 μg E2ZJ was sufficient to induce protective antibodies against CSFV in piglets and conferred extensive protection against the homologous and heterologous strains of CSFV. These results suggest that SPZJ-E2ZJ is a cost-effective and efficacious vaccine candidate against CSFV.

### 5.5. Cost-Effective E2 Protein Production Using a Plant Expression System

Compared with insect cells, plants are easy to grow, do not require specialized facilities, and production is easy to scale up. Thus, the use of E2 expressed in plants has been examined as a method to provide cost-effective protein production. Previously, CSFV E2 antigen was expressed in and purified from plant cells. The purified protein was shown to trigger specific immune responses in non-target animal models [138,139] and in pigs [140]. However, few data were available on the protective effect of a plant-made E2 antigen against CSFV challenge in pigs. Several recent studies, however, have presented data evaluating immunogenicity, safety, and efficacy of the plant-made E2 antigen against CSFV challenge in pigs.

Eun-Ju et al. showed that E2 fused with a cellulose-binding domain (CBD) could be expressed as a fusion protein at high level (0.7% of total soluble proteins) in transgenic *Arabidopsis thaliana* plants [141]. As an affinity tag for purification, CBD can be used for cost-effective, one-step purification. Consequently, the E2 fusion protein was purified in a single step with high yield and purity. The E2 protein is highly immunogenic in mice and could be recognized by CSFV-specific antibodies. In a later study, they reported the use of transgenic *Nicotiana benthamiana* plants for large-scale, cost-effective production of the plant-produced E2 fusion protein (ppE2) [142]. They demonstrated that ppE2 was effective in protecting piglets from CSFV challenge following prime-boost vaccination. In addition, they found that a single-dose vaccination (100 μg of ppE2) took 11 days to confer complete protection against CSFV challenge. Furthermore, they proposed that the CBD, which was highly immunogenic can function as a DIVA marker for the vaccination. Likewise, another research group employed an *Agrobacterium*-mediated transient expression platform in *N. benthamiana* for expression of E2 and formulated the purified antigen in novel oil-in-water emulsion adjuvants [143]. A single-dose vaccination (200 μg of E2 protein) induced a strong neutralizing antibody response, providing complete protection in pigs against CSFV challenge. Park et al. further investigated the production of a fusion of the CSFV E2 protein and the pFc2 domain of porcine IgG, which was designated as pmE2:pFc2, in transgenic *N. benthamiana* [144]. Fusing proteins to the Fc domain could enhance the solubility and half-life of an antigenic protein [145,146]. The results showed that purification of the pmE2:pFc2 protein was at high yield and low cost. Vaccination with the pmE2:pFc2 protein generated a CSFV-neutralizing antibody response in mice and pigs.

Taken together, the plant expression system is expected to provide single-dose potent CSFV subunit DIVA vaccines at a low manufacturing cost. Alternatively, to further reduce manufacturing costs, oral vaccine formulations using non-processed or semi-processed plant tissue expressing the E2 antigen could also be tested.

### 5.6. Surface Display of E2

To optimize E2 production in the yeast *Pichia pastoris*, the use of chicken-lysozyme signal peptide (cSIG) together with a multi-copy integration strategy was investigated in a recent study. It was shown that this strategy dramatically improved E2 production up to 30.72-fold [147]. Later, the researchers investigated the use of GEM particles for surface display of E2 expressed in *Pichia pastoris.* They finally generated the E2-Spy-PA-GEM complex [148]. The results showed that E2-Spy-PA-GEM induced higher anti-E2 and neutralizing antibody responses than E2-Spy in mice, indicating that the GEM-loaded particles exhibited enhanced immunogenicity compared with E2 alone. In another study, the E2 protein was expressed in *Escherichia coli.* Gold nanoparticles (AuNPs) were used to display E2 protein, generating E2-conjugated AuNPs (E2-AuNPs) [149]. The results showed that E2-AuNPs induced relatively higher antibody levels, lymphocyte proliferation indices, and IFN-γ and IL-10 cytokine levels than E2 or AuNPs in mice. Thus, the display of E2 protein using GEM particles or AuNPs has produced encouraging preliminary results in mice, and further data in pigs are needed to evaluate the efficacy of the new DIVA vaccine candidates.

## 6. Alternative Approaches

Other DIVA vaccines, such as peptide vaccines and DNA vaccines have also been generated against CSFV. Studies with these candidates were reviewed previously [32]. Briefly, peptide vaccine candidates generally contained either one peptide or a mixture of different peptides covering different parts of the antigenic domains of the E2. However, none of the evaluated candidates were able to confer complete protection upon CSFV challenge. As for DNA vaccines, all the vaccine candidates described so far are based on plasmid constructs that express the E2 protein. The high costs associated with DNA preparation and the inefficient delivery make DNA vaccines economically non-viable for CSFV control. Thus, these vaccines have not been under investigation for use since that review.

## 7. Final Remarks

CSF is a swine disease that continues to pose a threat to pig production worldwide. Vaccination with safe and highly efficacious live attenuated vaccines has been practiced for decades to control the disease and has paved the way to successful eradication of CSF from several countries. While these vaccines generally have outstanding properties in terms of onset and duration of immunity, their main drawback is the lack of DIVA concept. As the first generation of DIVA vaccines, commercial E2 subunit vaccines are highly safe but lack some efficacy and transferability to an oral formulation. Therefore, several research groups have sought to develop next-generation DIVA vaccine candidates that ideally meet all requirements regarding safety, efficacy, DIVA potential, and marketability (see Table 1). So far, great progress has been made in live attenuated DIVA vaccines, viral vector vaccines, and subunit vaccines. However, the DIVA diagnostics still show some room for improvement. Together with good surveillance and biosecurity, the vaccine candidates are promising for CSF control and eradication in endemic situations. In addition, these attempts also provide many feasible strategies and methods for the development of DIVA vaccines against other diseases.

## Figures and Tables

**Table 1:** Candidate differentiability of infected from vaccinated animals (DIVA) vaccines against Classical swine fever virus (CSFV) published in the last few years.

Type of Vaccine	Construct	Presented Data	Publication(s)
Live attenuated DIVA vaccine (chimeric pestivirus)	Chimeric pestivirus of CSFV vaccine strain LOM and KD26 BVDV strain (Flc-LOM-BE^rns^)	Immune responses, protect fetuses from transplacental transmission after challenge of pregnant sows with CSFV strain (YC11WB) in different pregnancy period (early, mid- or late)	[66]
Live attenuated DIVA vaccine (chimeric pestivirus)	Chimeric pestivirus of CSFV strain Alfort-p447 and pestiviruses distantly related to CSFV (Ra, Pro and RaPro)	Solid protection against clinical signs of CSF at 28 days post single vaccination Improved DIVA capacity	[67]
Live attenuated DIVA vaccine (Recombinant deletion or mutation DIVA vaccines)	Double antigenic marker vaccine with a positive marker (Flag in the E1) and a negative marker in the E2 (FlagT4Gv)	Induce immunity against challenge as early as 3 days post single vaccination	[68,69]
Live attenuated DIVA vaccine (Recombinant deletion or mutation DIVA vaccines)	C-strain with ^122^P of E2 mutated to A (rHCLV-E2P122A)	Retain the characteristics in both rabbits and pigs consistent with that of C-strain, induce anti-CSFV nAbs	[75]
Vector vaccine	Bivalent vaccine with a swinepox virus vector expressing CSFV E2 (rSPV-E2)	Induce a substantial CSFV-specific neutralizing antibody response in pigs. Provide clinically protection against CSFV challenge at 35 days post-primary immunization	[80,81]
Vector vaccine	Bivalent vaccine with a marker pseudorabies virus vector expressing CSFV E2 (rPRVTJ-delgE/gI-E2 and rPRVTJ-delgE/gI/TK-E2)	Induce detectable anti-PRV and anti-CSFV neutralizing antibodies. Complete protection against the lethal challenge with either the PRV TJ strain or the CSFV Shimen strain	[84,85,86]
Vector vaccine	Bivalent vaccine with a marker pseudorabies virus vector expressing CSFV E2 (JS-2012-ΔgE/gI-E2)	Provide protection to piglets against lethal challenge of PRV variant and CSFV after single vaccination	[88]
Vector vaccine	Bivalent vaccine with a porcine reproductive and respiratory syndrome virus vector expressing CSFV E2 (rPRRSV-E2)	Fully protect pigs from lethal challenge with HP-PRRSV and CSFV post single vaccination. PRRSV MDAs do not interfere with the immunization. Confers long-term immunity against HP-PRRSV and CSFV for at least 20 weeks and up to 24 weeks after vaccination.	[89,90,91]
Vector vaccine	Newcastle disease virus vector expressing CSFV E2 and E^rns^ (rNDV)	No shedding. Immune responses.	[102]
Vector vaccine	Baculovirus vector expressing CSFV E2 (BV-VSVG-ED-pFc-CMV-S/P-E2)	Immune responses.	[106]
Subunit vaccine	Recombinant CSFV E2 expressed in baculovirus system (TWJ-E2^®^)	Provide full protection against the highly virulent genotype 1.1 reference Shimen strain and genotype 2 field strains after two-dose vaccination.	[121,122]
Subunit vaccine	CSFV E2 antigen fused to the extracellular domain of porcine CD154 expressed in HEK 293 cell line (E2-CD154)	Protect full protection from challenge as early as 7 days post a single dose vaccination of pigs. Both doses (50 μg or 75 μg) were equally effective. Two-dose vaccination protected the fetuses from transplacental transmission after challenge of pregnant sows with a high dose of a highly virulent CSFV.	[127,128,129]
Subunit vaccine	CSFV E2 and porcine IFN-γ proteins co-expressed in baculovirus system	Significantly enhanced specific protective immune response in weaning piglets.	[131]
Subunit vaccine	E2 protein of C-strain expressed in baculovirus system, oil-in-water emulsion adjuvant KNB (KNB-E2)	Pigs vaccinated with a single KNB-E2 dose containing 25 μg of recombinant CSFV glycoprotein E2 were protected from clinical symptoms of CSF.	[132,134]
Subunit vaccine	E2 protein of a local genotype 1.1 CSFV isolate VN91 expressed in baculovirus system (VN91-E2)	Effectively stimulate protective immunity against CSFV with a two-dose vaccination. Protect pigs from different genotype (1.1, 2.1, and 2.2) CSFV challenge which is circulating in Vietnam	[136]
Subunit vaccine	E2 sequence identified from an epidemic strain of Zhejiang (E2ZJ) combined with signal sequence SPZJ expressed in baculovirus system (SPZJ-E2ZJ)	A single dose of 5 μg E2ZJ was sufficient to induce protective antibody against CSFV in piglets and conferred broad protection against the homologous and heterologous CSFV strains.	[137]
Subunit vaccine	Expression of E2 in transgenic rice calli (rE2-TRCs)	Immune responses in mice and pigs after oral vaccination	[140]
Subunit vaccine	E2 fused with a cellulose-binding domain (CBD) expressed in transgenic *Arabidopsis thaliana* plants	Highly immunogenic in mice and could be recognized by CSFV-specific antibodies.	[141]
Subunit vaccine	E2 fusion protein (ppE2) expressed in transgenic *N. benthamiana* plants	Provide effective protection against CSFV infection following prime-boost vaccination of piglets. A single-dose ppE2 vaccination with 100 μg requires 11 days post-vaccination to confer full protection from CSFV infection.	[142]
Subunit vaccine	E2 expressed using an *Agrobacterium*-mediated transient expression platform in *N. benthamiana*	Provide complete protection in challenged pigs, even after single-dose vaccination (200 μg E2 protein).	[143]
Subunit vaccine	Fusion of the CSFV E2 protein and the pFc2 domain of porcine IgG expressed in transgenic *N. benthamiana* (pmE2:pFc2)	Vaccination of mice and pigs with the pmE2:pFc2 protein generated a neutralizing antibody response against CSFV.	[144]
Subunit vaccine	GEM particles for surface display of the E2 glycoprotein expressed in the *Pichia pastoris* (E2-Spy-PA-GEM)	Higher anti-E2 and neutralizing antibody responses than E2-Spy in mice.	[148]
Subunit vaccine	AuNPs for surface display of the E2 protein expressed in *E. coli* (E2-AuNPs)	Induced relatively higher antibody levels, lymphocyte proliferation index, IFN-γ and IL-10 cytokines than those of E2 or AuNPs group in mice.	[149]

## Data Availability

No new data were created or analyzed in this study. Data sharing is not applicable to this article.
